# Diagnostic accuracy for different strategies of image-guided breast intervention in cases of nonpalpable breast lesions

**DOI:** 10.1038/sj.bjc.6601559

**Published:** 2004-02-03

**Authors:** R M Pijnappel, M van den Donk, R Holland, W P Th M Mali, J L Peterse, J H C L Hendriks, P H M Peeters

**Affiliations:** 1 Department of Radiology, Martini Hospital, Locatie van Swieten, PO Box 30033, 9700 RM Groningen, The Netherlands; 2 Department of Epidemiology, University Medical Centre Nijmegen, PO Box 9101, 6500 HB Nijmegen, The Netherlands; 3 National Expert and Training Centre for Breast Cancer Screening, University Medical Centre Nijmegen, PO Box 9101, 6500 HB Nijmegen, The Netherlands; 4 Department of Radiology, University Medical Centre Utrecht, PO Box 85500, 3508 GA Utrecht, The Netherlands; 5 Department of Pathology, The Netherlands Cancer Institute/Antoni van Leeuwenhoek Hospital, Plesmanlaan 121, 1066 CX Amsterdam, The Netherlands; 6 Julius Centre for General Practice and Patient Oriented Research, Utrecht University Medical School, PO Box 85500, 3508 GA Utrecht, The Netherlands

**Keywords:** Breast biopsy, core biopsy, FNA

## Abstract

To find out whether ultrasound-guided fine-needle aspiration (FNA) and ultrasound and stereotactic-guided large core needle biopsy (LCNB) are reliable alternatives to needle-localised open breast biopsy (NLBB) in daily practice, we performed a retrospective study and evaluated the validity of these methods. In all, 718 women with 749 nonpalpable breast lesions from three Dutch Hospitals were included, and the validity of the various methods for diagnosis was assessed. This was carried out according to a method described by Burbank and Parker for evaluating the quality of an image-guided breast intervention. We compared our results with the outcome of the COBRA study. Overall, all diagnostic strategies (NLBB, FNA, LCNB ultrasound and stereotactic guided) show comparable agreement rates. However, the miss rates differ: 2% for NLBB, 3% for COBRA (LCNB in study setting), 5% for FNA and 8–12% for LCNB in practice. Fine-needle aspiration was nonconclusive in 29%, and shows an overestimation for DCIS in 9%. The DCIS underestimate rate in NLBB was 8%. For the assessment of lesions consisting of microcalcifications only and to exclude malignancy in all other lesions, a 14-gauge needle should be used. Ultrasound-guided intervention can be performed in a large percentage of nonpalpable lesions. Lesions consisting only of microcalcifications on mammography need special attention.

Needle-localised open breast biopsy (NLBB) is considered the gold standard procedure for the diagnosis of nonpalpable breast lesions (NPBL) (Jackman and Marzoni, 1997). Needle-localised open breast biopsy is a surgical procedure with high costs and for most of the patients, is a traumatic experience. In the past few decades, less traumatic and cost-saving nonoperative image-guided techniques, such as fine-needle aspiration (FNA) and large-core needle biopsy (LCNB), have been advocated as an alternative to surgery to obtain material for a microscopic diagnosis. For guidance, ultrasound or stereotaxis are advocated.

Recently, a large prospective study on stereotactic-guided LCNB was performed in the Netherlands to evaluate the diagnostic accuracy of this method (COBRA study) (Verkooijen, 2002). This study included 973 patients with 1029 nonpalpable breast lesions and showed a sensitivity rate of 97% and a specificity rate of 99% for stereotactic-guided LCNB. These figures are comparable to NLBB (Jackman and Marzoni, 1997). In the Dutch study, the stereotactic LCNB was always performed on a dedicated prone table, in an optimal setting. The dedicated equipment appears to be highly reliable but has the disadvantage of high initial expense. Therefore, it is not widely available in the Netherlands. The other alternatives for NLBB, such as stereotactic-guided LCNB with upright mammography machine and add on digital attachment, ultrasound-guided FNA and LCNB are more widely available. To find out whether FNA (ultrasound-guided) and LCNB (ultrasound and stereotactic-guided) are reliable alternatives to NLBB in daily practice, a retrospective study on the diagnostic accuracy of these different techniques was performed in three hospitals in the Netherlands.

The aim of this study was to evaluate the method of choice for intervention and type of guidance with respect to the mammographic characteristics of the lesion.

We present the results of 718 women with 749 nonpalpable breast lesions diagnosed with NLBB, FNA and LCNB in three hospitals in the Netherlands, and compare these results with the outcome of the COBRA study (Verkooijen, 2002).

## PATIENTS AND METHODS

In this study, all patients were included with a mammographic suspect, nonpalpable lesion in a defined calendar period, who were referred for diagnostic work-up at three different hospitals in the Netherlands: the University Medical Centre Utrecht (UMCU), the University Medical Centre Nijmegen (UMCN) and the Antoni van Leeuwenhoek Hospital Amsterdam (AvL). These three hospitals were chosen because of their different strategies and their large experience in diagnostic work-up for patients referred with nonpalpable breast lesions. Patients were identified by means of the hospital registry. We used the central computer systems of the hospital registrations to obtain the records of all patients who underwent an NLBB or an image-guided breast intervention. A total of 202 women were identified at the UMCU (January 1996–December 1999), 237 women at the UMCN (January 1996–December 1999) and 279 women at the AvL (January 1997–December 1999). These 718 women had 749 nonpalpable mammographic suspect lesions. The records of all radiological and pathological breast examinations were collected. The following data were abstracted: patient characteristics, type of imaging technique used at first examination, mammographic lesion characteristics, ultrasound lesion characteristics, biopsy method (NLBB, FNA, LCNB), pathological lesion characteristics and imaging follow-up results.

Needle-localised open breast biopsy was performed as a primary diagnostic procedure as well as a secondary diagnostic procedure for confirmation after FNA or core biopsy. In cases of NLBB, the needle was placed with ultrasound guidance or X-ray guidance using a coordinate grid or stereotaxis.

Fine needle assay was performed with a 22-gauge needle and a 10-ml syringe. Adequate needle position was controlled by real-time visualisation of the tip-echo in the lesion.

Stereotactic-guided LCNB was performed at the UMCN with a conventional add-on device and at the UMCU with a digital dedicated prone table. At the UMCN, for all LCNB an 18-gauge needle was used. The other hospitals used a 14-gauge long throw needle. The number of passes varied from two to three with ultrasound guidance. With stereotactic guidance, the number of passes varied from two to three at the UMCN, but at the UMCU a minimum of five passes were taken.

We assessed the validity of NLBB, FNA and LCNB (ultrasound and stereotactic). This was carried out according to the method of Burbank and Parker (1998), which uses a four by four table, instead of a two by two table for the analysis. (Table 1

**Table 1:** Adjusted Burbank & Parker method for the evaluation of results of image-guided intervention and histological findings at surgical excision

	**Histologic findings of surgical excision**				
**Image-guided intervention**	**Nonconclusive**	**Benign**	**High risk**	**DCIS**	**Invasive carcinoma**
Nonconclusive		NC	NC	NC	NC
					
Benign		A	U (agreement)	U (miss)	U (miss)
High risk (histology)		O (agreement)	A	U (HR underestimate)	U (HR underestimate)
DCIS		O	O	A	U (DCIS underestimate)
Invasive carcinoma		O (agreement)	O (agreement)	O (agreement)	A
Malignancy on cytology		O (overestimate)	O (overestimate)	O (DCIS overestimate)	A

Agreement=the patient management was correct on behalf of the pathology findings on core biopsy; miss=core biopsy missed the clinically relevant malignancy (*in situ* or invasive); HR underestimate=although a high-risk lesion was found on core biopsy and the appropriate clinical management was taken (surgical biopsy), the malignancy (*in situ* or invasive) was missed on core; DCIS underestimate=in the surgical specimen for the treatment of DCIS found on core biopsy, an invasive component was discovered; Overestimate=although malignant cells were found on FNA, no invasive carcinoma was found in the surgical specimen.NC=nonconclusives; A=agreements; O=overestimates; U=underestimates.

) In the latter, much important clinical information is lost, while the former allows not only the distinction between benign and malignant lesions but also to include in the analysis pathologic findings such as atypical ductal hyperplasia (ADH), ductal carcinoma *in situ* (DCIS) or infiltrating breast cancer. Atypical ductal hyperplasia lesions are often small and therefore the identification of ADH in a surgical specimen is nearly always an incidental finding without therapeutic consequences (Tavassoli, 1998). Nevertheless, ADH found in a core biopsy specimen has clinical consequences because in a high percentage it is associated with malignancy (Liberman *et al*, 1995). The difference between DCIS and invasive breast cancer has clinical consequences. If a lesion was diagnosed with LCNB as ADH, DCIS or invasive breast cancer, but the surgical excision showed a lower degree of pathology, it is more appropriate to regard this as an agreement rather than an overestimate. It happens that an accurately diagnosed lesion at needle sampling is not again identified at surgical biopsy, because the lesion was already totally removed at needle sampling, or the lesion was not adequately removed at surgery.

We had to make an adjustment of the four by four table for FNA. With FNA, it is not possible to determine whether a malignancy is invasive or not. In our model, when the FNA was reported malignant, it is regarded as an agreement only if the histological diagnosis after surgery was invasive breast cancer. If the histological diagnosis after surgery was DCIS, we regarded it a ‘DCIS overestimate’. In the table, we added a column ‘nonconclusive’ to be able to evaluate the number of insufficient samples. Since we used retrospective data from on-going clinical practice, the NLBB (gold standard) was not available for all patients. For these patients, we used follow-up data to assume the absence of malignancy. In case the follow-up was too short, we assumed correctness of the primary diagnostic procedure to estimate the upper border of the rates (sensitivity analysis).

Data were administered and analysed using the Statistical Package for Social Sciences 8.0 (SPSS Inc., Chicago, IL, USA).

## RESULTS

In Table 2

**Table 2:** Characteristics of the study population

	**Total population**	**UMCU**	**UMCN**	**AvL**
Number of patients	718	202	237	279
Number of lesions	749	211	255	283
				
Age (range)	54.7 (21–85)	52.8 (24–85)	54.8 (26–84)	55.9 (21–83)
				
Reason for examination				
Screen-detected lesions	208 (27.8)	56 (26.5)	90 (35.3)	62 (21.9)
Family history of breast cancer	98 (13.6)	27 (13.4)	49 (20.7)	22 (7.9)
History of breast cancer	136 (18.9)	33 (16.3)	39 (16.5)	64 (22.9)
Other a	155 (21.6)	58 (28.7)	54 (22.8)	43 (15.4)
Unknown	152 (21.2)	37 (18.3)	23 (9.7)	92 (32.9)
				
Mammography	729 (97.3)	208 (98.6)	245 (96.1)	276 (97.5)
Only microcalcifications	223 (30.6)	63 (30.3)	73 (29.7)	87 (31.5)
Density with microcalcifications	85 (11.7)	18 (8.7)	33 (13.5)	34 (12.3)
Density	252 (34.5)	72 (34.6)	81 (33.1)	99 (35.9)
Spiculated density	96 (13.2)	22 (10.5)	32 (13.1)	42 (15.3)
Focal asymmetry	3 (0.4)	1 (0.5)	2 (0.8)	0
Architectural distortion	12 (1.6)	1 (0.5)	9 (3.7)	2 (0.7)
Not visible	56 (7.7)	31 (14.9)	15 (6.1)	10 (3.6)
Unknown	2 (0.3)	0	0	2 (0.7)
				
Ultrasonography				
Number (% of lesion per clinic)	567 (75.7)	135 (64.0)	202 (79.2)	230 (81.3)
Solid	410 (72.3)	110 (81.5)	128 (63.4)	172 (74.8)
Both solid and cystic	16 (2.8)	6 (4.4)	7 (3.5)	3 (1.3)
Not described	16 (2.8)	1 (0.7)	8 (3.9)	7 (3.0)
Not visible	125 (22.1)	18 (13.4)	59 (29.2)	48 (20.9)
				
Radiologic diameter of lesion				
No. of cases known (%)	450 (60.1)	84 (39.8)	169 (66.3)	197 (69.6)
mm (s.d.)	13.9 (10.8)	10.9 (5.3)	16.3 (12.7)	13.0 (10.3)
				
Primary diagnostic intervention				
Diagnostic surgery	316 (42.2)	79 (37.4)	129 (50.6)	108 (38.2)
FNA ultrasound guided	242 (32.3)	72 (34.1)	0	170 (60.1)
LCNB ultrasound guided	128 (17.1)	21 (10.0)	102 (40.0)	5 (1.7)
LCNB stereotactic guided	63 (8.4)	39 (18.5)	24 (9.4)	0
				
Malignant lesions (histology)^b^	378 (50.4)	84 (39.8)	130 (50.9)	164 (57.9)
DCIS	102 (27.0)	26 (31.0)	34 (26.2)	42 (25.6)
Invasive carcinoma	276 (73.0)	58 (69.0)	96 (73.8)	122 (74.4)

a Includes: palpable lesion somewhere else in the breast(s), follow-up for benign condition in the past, pain, hormone replacement therapy.

b Histology report from surgical specimen (‘Gold standard’)

, the characteristics of the patients and the results of the radiological examinations are presented. The mean age of the patients was 54.7 years (range 21–85 years) In all, 208 (27.8%) lesions were screen detected. Of all 749 lesions, 729 (97.3%) were examined with mammography; 223 (30.6 %) showed only microcalcifications, 433 (59.4 %) showed a mass (including densities, densities with microcalcifications and spiculated masses) and 56 (7.7%) were not visible at mammography. The mean diameter of the lesions (according to the radiological examinations) was 13.9 mm. Malignancy was diagnosed in 378 (50.4%) lesions, DCIS in 102 out of 378 (27.0%) and invasive carcinoma in 276 out of378 (73.0%).

A primary surgical diagnostic approach was performed in 316 out of 749 (42.2%) cases. In 176 cases, the lesion consisted of microcalcifications only (55.6%). In four primary surgical procedures, the amount of tissue removed was not sufficient for reliable diagnosis (1.3%). The procedure failed six times because the lesion was not removed (2%). Of these six lesions, two were removed during a second operation and four were followed radiologically. The results of the 312 lesions were: 129 benign, 23 high-risk (ADH, lobular carcinoma *in situ*), 74 DCIS and 86 invasive carcinoma. Six patients who were initially diagnosed as having DCIS also showed invasive carcinoma on therapeutic excision (DCIS underestimate rate 8%).

Fine-needle aspiration was followed by NLBB in 148 out of 242 lesions (61.2%). The results are presented in Table 3

**Table 3:** Comparison of cytologic findings of FNA to histological findings of surgical excision

		**Histologic findings of surgical excision**					
	**No surgical confirmation**^a^	**Nonconclusive**	**Benign**	**High risk**	**DCIS**	**Invasive carcinoma**	**Total**
Cytologic findings							
Nonconclusive	35		18 (NC)	2 (NC)	2 (NC)	13 (NC)	70
Benign	54		17 (A)		1 (M)	4 (M)	76
	1						1
Malignancy	4		2 (O)		8 (O DCIS)	81 (A)	95
							
Total	94	0	37	2	11	98	242

a No surgical confirmation at the hospital where the FNA was performed. NC=nonconclusives; A=agreements; M=misses; O=overestimates.

. Fine-needle aspiration was nonconclusive in 70 out of 242 cases (29%). There were 98 agreements, 10 overestimates and five misses. For 94 lesions (38.8%), NLBB was not available. Of these 94, 17 cases were followed radiologically after a nonconclusive FNA (3–33 months; mean 13.8) and one carcinoma was found on follow-up. A total of 22 lesions with a benign result on FNA were followed radiologically (4–38 months; mean 13.4), so far without any suspect abnormalities. Of these 22 cases, 21 were classified as BIRADS category 3; one was classified as BIRADS 4. For 31 lesions, the period between FNA and planned mammographical and clinical follow-up was too short. The remaining 24 lesions were not planned for NLBB for various reasons. (Cyst: seven; refused therapy: one; malignant tumour (no breast cancer) found elsewhere: two; treatment in another hospital: three; lost for follow-up: nine; LCNB with benign result without follow-up: two).

For 89 out of 128 (69.5%) lesions that were examined with LCNB under ultrasound guidance, a surgical excision was performed. The results are shown in Table 4

**Table 4:** Comparison of histologic findings of LCNB US-guided to histological findings of surgical excision

		**Histologic findings of surgical excision**					
	**No surgical confirmation**^a^	**Nonconclusive**	**Benign**	**High risk**	**DCIS**	**Invasive carcinoma**	**Total**
LCNB US-guided							
Nonconclusive	1		1 (NC)			3 (NC)	5
							
Benign	34		17 (A)	2 (A)	2 (M)	6 (M)	61
							
High risk	1			1 (A)		1 (U HR)	3
							
DCIS					5 (A)	3 (U DCIS)	8
							
Invasive carcinoma	3					48 (A)	51
							
Total	39	0	18	3	7	61	128

a No surgical confirmation in the hospital where the US-guided LCNB was performed. US-guided=ultrasound guided.NC=nonconclusives; A=agreements; M=misses; U=underestimates.

. There were 73 agreements, four underestimates and eight misses. The other four were nonconclusive on LCNB. The gold standard procedure was not available in 39 lesions (30.5%). Of these 39, a radiological follow-up was available in 14 cases with benign result (2–34 months; mean 17.5). No carcinoma was found at follow-up. For 14 lesions, the period between LCNB and planned mammographical and clinical follow-up was too short to have follow-up data yet. The reasons for the remaining 11 were: cyst: one; metastasis: one; lost to follow-up: three; LCNB with benign result without follow-up: six.

For lesions diagnosed by stereotactic LCNB, NLBB was available in 54 out of 63 cases (85.7%). (Table 5

**Table 5:** Comparison of histologic findings of LCNB stereotactic guided to histological findings of surgical excision

		**Histologic findings of surgical excision**					
	**No surgical confirmation**^a^	**Nonconclusive**	**Benign**	**High risk**	**DCIS**	**Invasive carcinoma**	**Total**
LCNB stereotactic							
Nonconclusive			1 (NC)		1 (NC)		2
							
Benign	8		10 (A)	1 (A)	3 (M)		22
							
High risk			2 (A)	1 (A)	2 (U HR)		5
							
DCIS					8 (A)	3 (U DCIS)	11
							
Invasive carcinoma	1					22 (A)	23
							
Total	9	0	13	2	14	25	63

a No surgical confirmation in the hospital where the stereotactic-guided LCNB was performed.NC=nonconclusives; A=agreements; M=misses; U=underestimates.

) There were 44 agreements, five underestimates and three misses. All the misses with stereotactic-guided LCNB were found in the group of lesions consisting of microcalcifications only. The other two were nonconclusive on LCNB. For nine lesions (14.2%), a gold standard procedure was not available. The reasons were treatment in another hospital: one, lost for follow-up: two. In four cases with a benign result, radiological follow-up was available (2–31 months; mean 20.5). No carcinoma was found at follow-up. For two lesions, the period between LCNB and planned follow-up was too short.

In Table 6

**Table 6:** Agreement rate, underestimate and overestimate rates for the different primary diagnostic procedures

	**Surgical biopsy**	**FNA US-guided**	**LCNB US-guided**	**LCNB stereotactic**	**COBRA study**
**Needle (gauge)**		**22G**	**18G+14G**	**14G**	**14G**
*n*=	316	148	89	54	858
Nonconclusive (%)	1.2	29	4	3	1.5
Agreement rate (%)	Not applicable	87	86	85	93
Miss rate (%)	2	5	12	8	3
Sensitivity (%)	98	95	88	92	97
High risk underestimate rate (%)^a^	9	Not applicable	33	40	23
DCIS underestimate rate (%)^b^	8	Not applicable	37	27	17
DCIS overestimate rate (%)^c^	Not applicable	9	Not applicable	Not applicable	Not applicable

a High-risk underestimate is defined as the finding of ADH, lobular carcinoma *in situ* without malignancy in the diagnostic material, but with carcinoma (*in situ* or invasive), in the surgical specimen

b DCIS underestimate is defined as the finding of DCIS without invasive carcinoma in the diagnostic material, but with invasive carcinoma in the therapeutical specimen

c DCIS overestimate is defined as the finding of malignant cells on cytology and DCIS without invasive carcinoma in the surgical specimen. US-guided=ultrasound guided.

, the agreement, underestimate and miss rates are presented and compared with the results from the COBRA study.

Sensitivity analysis was used to quantify the impact of patient selection for additional NLBB. The miss rate and sensitivity rate remains the same, since we assume them to be nonmalignant. Agreement rates increase: for FNA from 87 to 91%, for ultrasound-guided LCNB from 86 to 90% and for stereotactic-guided LCNB from 85 to 87%.

## DISCUSSION

Overall, all diagnostic strategies (NLBB, FNA, LCNB ultrasound and stereotactic guided) show comparable agreement rates. However, the miss rates differ: 2% for NLBB, 3% for COBRA (LCNB in study setting), 5% for FNA and 8–12% for LCNB in practice, respectively.

Ductal carcinoma *in situ* underestimate (the finding of DCIS without invasive carcinoma in the diagnostic material, but with invasive carcinoma in the therapeutically specimen) varies from 8% (NLBB) to 37% (LCNB ultrasound guided). The difference in treatment of *in situ* and invasive carcinoma with respect to the axilla makes this a serious clinical problem. Previous studies on the underestimation of DCIS in a diagnostic surgery show figures of 16 and 17% in a smaller series of patients (3 out of 19 and four out of 24) (Thompson *et al*, 1991; Tartter *et al*, 1997). Our figure of six out of 74 (8%) confirms the fact that DCIS underestimation is also a problem in diagnostic NLBB. Ductal carcinoma *in situ* underestimation of LCNB has often been considered as a specific disadvantage of this technique, but every form of sampling, whether surgical or image-guided minimal invasive, deals with this problem. Therefore, the often results with respect to DCIS underestimation in nonsurgical biopsy studies have to be compared to the figures of surgical biopsies.

High-risk underestimate (the finding of ADH, lobular carcinoma *in situ* without malignancy in the diagnostic material, but with carcinoma (*in situ* or invasive), in the surgical specimen) varies from 9% (NLBB) to 40% (LCNB stereotactic guided). In the literature, figures of high-risk underestimate for 14-gauge needle biopsy range from 14 to 58%. (Jackman *et al*, 1999; Verkooijen *et al*, 2000) It is difficult to characterise the nonsurgical high-risk group as a whole. Most frequently, this group is being used for lesions with a microscopic pattern for which there is no consensus in classification or in cases where final classification is difficult or even impossible because of the small amount of tissue. Therefore, high risk is frequently being used as an ‘escape category’ for lesions difficult to diagnose (e.g. atypia at FNA and ADH at core biopsy). In case of a lesion classified as high risk, a larger amount of tissue is needed for final diagnosis.

In understanding the computed estimates, one should keep in mind two aspects of patient selection. First, the selection of primary diagnostic procedure in relation to the imaging characteristics, and second patient selection for the surgical histopathologic confirmation of the primary diagnostic procedure.

Fine-needle aspiration was only used with ultrasound guidance, and therefore in selected cases. This selection was based on the imaging characteristics of the lesion. Only 5.3% of the lesions diagnosed with FNA consisted only of microcalcifications. For the whole population, this figure was 30.6%. So, FNA was mainly used in cases of lesions not just consisting of microcalcifications. For the interpretation of the miss rate figures this is important to realise. It has been reported that miss rates are higher in lesions consisting of microcalcifications (Lifrange *et al*, 1997). Fine-needle aspiration shows a high percentage of nonconclusive results (29%). This is a well-known major disadvantage of the technique. The reported rates for insufficient specimen with ultrasound-guided FNA vary from 0 to 38% for nonpalpable lesions (Klijanienko *et al*, 1998). The success of FNA is operator dependent (Masood, 1998). The best results have been reported with an experienced operator performing the aspirations. A multicentre clinical trial to evaluate FNA for nonpalpable lesions performed by multiple operators was terminated early because of the high rate of insufficient samples (Pisano *et al*, 1998). Comparison of the two institutions where FNA was performed in our study shows a percentage of nonconclusive samples of 21.8% at the AvL and of 45.8% at the UMCU. At the AvL, FNA is performed by a small group of radiologists and dedicated cytopathologists. The results at the AvL compare well with previous results (Ciatto *et al*, 1997). At the UMCU, the aspiration is performed by a large group of radiologists and residents. These high rates of insufficient samples as reported from the UMCU make its use impractical in a clinical setting, and supports the operator dependence as mentioned above. Another well-known problem of FNA is the fact that it is not capable of differentiating between *in situ* and invasive carcinoma in this study, resulting in a DCIS overestimation rate (the finding of malignant cells on cytology, and DCIS without invasive carcinoma in the surgical specimen) of 9%. As mentioned above, the difference in treatment of *in situ* and invasive carcinoma with respect to the axilla makes this a serious clinical problem.

For ultrasound-guided LCNB, the lesion selection based on the mammographic image was even more obvious. Only two cases (1.6%) consisted of only microcalcifications on mammography. Despite this selection, ultrasound-guided LCNB shows a relatively high miss rate of 12%. The result of ultrasound-guided LCNB in our study differs from the results in the literature on this subject. One study showed almost an equal accuracy with ultrasound-guided LCNB compared to NLBB, using a 14-gauge needle and a total of four to five passes (Parker *et al*, 1993). The 18-gauge needle with two passes in our study was responsible for six of the eight misses. This technique is definitely inferior to the one Parker described and the one that was used in the COBRA study. There is a discussion in the literature about whether an 18-gauge needle is acceptable for ultrasound-guided LCNB (Cardenosa, 1999). With respect to our figures, caution is warranted.

In stereotactic-guided LCNB, no selection was made. Half of the lesions consisted of microcalcifications only (50.7%). All three misses with stereotactic-guided LCNB were found in the group of lesions consisting of only microcalcifications. If we compare the two institutions where stereotactic-guided LCNB was performed, there is a clear difference in outcome. This is probably due to the technique used (18-gauge two to three passes (UMCN) *vs* 14-gauge with a minimum of five passes (UMCU)). The 18-gauge needle with two or three passes was responsible for 66.6% of the misses and 50% of the underestimated high risks. Although the number of cases with the 18-gauge needle is small, the figures are disappointing. A previous study showed that a 14-gauge needle provides the most accurate diagnosis, compared to 16- and 18-gauge needles (Nath *et al*, 1995). Furthermore, the diagnostic sensitivity is improved by increasing the number of cores taken to six or more, particularly in women with mammographic microcalcifications of an equivocal nature (Rich *et al*, 1999). The limitation in the assessment of the group ‘microcalcifications only’ indicates that a larger volume of tissue is necessary for a reliable histopathalogic diagnosis. Therefore, in both North America and many European breast diagnostic centres, vacuum-assisted core biopsy is now extensively used. This technique has the ability to obtain more diagnostic material during percutaneous biopsy compared to 14-gauge core biopsy.

The second important aspect of patient selection in our study (different from the COBRA, where all patients received a surgical histopathologic confirmation) is the selection that was made by surgeons of patients who underwent an ‘additional’ NLBB in cases of a benign result. It is reasonable to assume that those patients for whom the surgeon did not have a secure feeling about the benign primary diagnosis were selected for NLBB. And therefore, those patients not selected for subsequent NLBB have a correct primary diagnosis. Taking this into account, we computed the maximum agreement rates.

In conclusion, we found that FNA has a very high percentage of nonconclusive results and has no place in the diagnosis of lesions consisting of only microcalcifications. For the assessment of lesions consisting of microcalcifications only and to exclude malignancy in all other lesions, 18-gauge needle core biopsy is unsuited. Ultrasound-guided intervention can be performed in a large percentage of nonpalpable lesions. In a study setting, the dedicated prone table used in combination with state-of-the-art assessment protocols shows the best results. Lesions only consisting of microcalcifications on mammography need special attention.
